# Coexistence of Primary Myelofibrosis and Paroxysmal Nocturnal Hemoglobinuria Clone with *JAK2* V617F, *U2AF1* and *SETBP1* Mutations: A Case Report and Brief Review of Literature

**DOI:** 10.3390/diagnostics11091644

**Published:** 2021-09-08

**Authors:** Sholhui Park, Min-Kyung So, Min-Sun Cho, Dae-Young Kim, Jungwon Huh

**Affiliations:** 1 Department of Laboratory Medicine, College of Medicine, Ewha Womans University, Seoul 07804, Korea; solheepark@ewha.ac.kr (S.P.); mkso79@gmail.com (M.-K.S.); 2 Department of Pathology, College of Medicine, Ewha Womans University, Seoul 07804, Korea; mcho1124@ewha.ac.kr; 3 Department of Internal Medicine, College of Medicine, Ewha Womans University, Seoul 07804, Korea; rubatokim@gmail.com

**Keywords:** case report, primary myelofibrosis, paroxysmal nocturnal hemoglobinuria, *JAK2* V617F, *U2AF1* mutation, *SETBPT1* mutation

## Abstract

Primary myelofibrosis (PMF) and paroxysmal nocturnal hemoglobinuria (PNH) are very rare diseases, respectively, and it is uncommon to have both diseases together. Mutational profiling using next-generation sequencing in PMF and PNH detected additional mutations associated with myeloid neoplasms, suggesting a step-wise clonal evolution. We present here a very rare case with PMF and PNH with *JAK2* V617F, *U2AF1* and *SETBP1* mutations at the time of diagnosis. The combination of these two diseases and three genetic mutations is difficult to interpret at once. (i.e., the sequence of these two clonal diseases or the time points of acquiring these mutations). Our report suggests that when diagnosing or treating patients with PMF, it is necessary to keep in mind that PNH may be present at the same time or sometimes new. The genetic mutations simultaneously found in this patient require further research to elucidate the clinical significance and their genetic associations fully.

## 1. Introduction

Primary myelofibrosis (PMF) is one of the clonal myeloproliferative neoplasms (MPN) characterized by megakaryocytic and granulocytic proliferation in the bone marrow, resulting in bone marrow fibrosis and extramedullary hematopoiesis. Three driver mutations of Janus kinase 2 (*JAK2*), calreticulin (*CALR*) and myeloproliferative leukemia virus oncogene (*MPL*) are well implicated in MPN. In PMF patients, *JAK2* V617F mutation is found in 55% to 65% of cases, while *CALR* and *MPL* mutations are observed in 25% to 30% of cases [1]. Through the wide use of next-generation sequencing (NGS) in clinical practices, in addition to the three driver mutations, other genetic mutations (i.e., *ASXL1*, *EZH2*, *SRSF2*, *U2AF1Q157* and *IDH1/2*, etc.) have been reported in MPN [2,3,4,5,6].

Paroxysmal nocturnal hemoglobinuria (PNH) is characterized by chronic hemolysis and increased risk of thrombosis, which are caused by elevated complement activation in the phosphatidylinositol glycan class A (PIGA) deficient cells due to somatic *PIGA* mutation in hematopoietic stem cells [7]. Furthermore, the application of NGS in PNH patients demonstrated that the mutations of myeloid neoplasm-related genes (i.e., *TET2*, *SUZ12*, *U2AF1*, *JAK2*, *ASXL1*, *DNMT3A*, etc.) could be acquired in addition to the *PIGA* gene mutation [8,9,10].

There have been a few reports of the concomitant MPN and PNH [11,12,13,14,15,16,17], whereas a close association between PNH and aplastic anemia (AA)/myelodysplastic syndrome (MDS) has been well known [18].

Here, we report a case with the coexistence of PMF and PNH harboring *JAK2*, *U2AF1* and *SETBP1* gene mutations.

## 2. Case Presentation

An 87-year-old male was transferred to our hospital with a complaint of general weakness and dyspnea on exertion for two years. He has been diagnosed with severe anemia, thrombocytopenia, splenomegaly and newly developed multiple lymphadenopathies. He had a 2-year history of receiving periodic blood transfusions for iron deficiency anemia and other comorbidities included hypertension and cerebrovascular disease. Because the patient was transferred from another hospital, it was difficult to obtain detailed information about past diseases and treatments other than blood transfusion.

**Figure 1:**
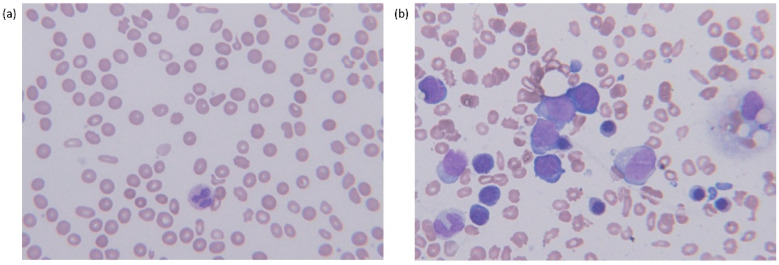
Morphologic features in peripheral blood and bone marrow aspirate of a primary myelofibrosis patient with PNH-clone, (**a**) peripheral blood showing pancytopenia with RBC poikilocytosis (Wright–Giemsa stain, ×400) and (**b**) bone marrow dilution with a few myeloblasts mixed with erythroid precursors (Wright–Giemsa stain, ×400).

The CBC findings of peripheral blood were as follows: hemoglobin (Hgb) 6.0 g/dL, reticulocyte percentage 2.58%, calculated reticulocyte production index (RPI) 0.54; platelet count 128 × 10^9^/L (Table 1). The white blood cell (WBC) count was 5.86 × 10^9^/L with neutrophils 56%, band neutrophils 4%, metamyelocytes 2%, myelocytes 7%, blasts 1%, lymphocytes 13%, monocytes 12%, eosinophils 1% and basophils 4%. Peripheral blood smear showed RBC poikilocytosis, such as teat drop cells, elliptocytes and acanthocytes (Figure 1a).

The other laboratory findings were as follows; elevated lactate dehydrogenase (LDH) 640 IU/L, decreased haptoglobin (lower than detection limit), elevated erythropoietin 189 mIU/mL. However, other laboratory tests for evidence of hemolysis showed normal results; normal bilirubin, normal aspartate aminotransferase (AST) and alanine aminotransferase (ALT), normal plasma free hemoglobin, no hemoglobinuria, negative hemosiderin and negative direct or indirect Coombs’ tests (Table 1). A PNH test was also performed based on the thrombocytopenia associated with hemolytic anemia and negative Coomb’s test. PNH clones were identified in 3.8% of RBCs, 48.6% of neutrophils and 77.2% of monocytes using flow cytometric analysis using CD59 for RBCs and FLAER & CD24 for WBCs. These results suggest extravascular hemolysis.

**Figure 2:**
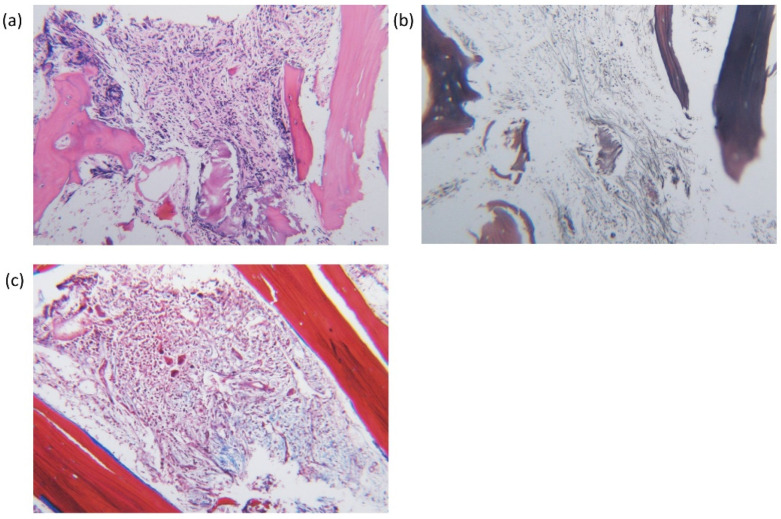
Diffuse myelofibrosis in bone marrow core biopsy, (**a**) hematoxylin and eosin stain (×100), (**b**) reticulin fibrosis by retuculin stain (×100) and (**c**) collagen fibrosis by Masson’s trichrome stain (×100).

Computed tomography (CT) and Positron Emission Tomography (PET)/CT scan showed splenomegaly, two masses in the inferior portion of the spleen, multiple lymphadenopathies and hypermetabolism in the bone marrow. Splenic or portal vein thromboses were not found. Splenic fine needle biopsy revealed extramedullary hematopoiesis. A bone marrow (BM) aspirate was diluted but showed clusters of myeloid precursors (Figure 1b). BM core biopsy revealed overt myelofibrosis with megakaryocytic hyperplasia and atypia (Figure 2). Conventional cytogenetic analysis showed normal karyotype, 46,XY. Molecular analysis using targeted NGS (Oncomine Myeloid Research Assay, Ion Torrent, Thermo Fisher Scientific) revealed *JAK2* V617F with 50.9% variant allele frequency (VAF). In addition, *U2AF1* and *SETBP1* missense mutations were identified: *U2AF1*, NM_001025203.1:c.470A > C, (p.Gln157Pro) with 44.7% of VAF; *SETBP1*, NM_015559.3:c.2608G > A, (p.Gly870Ser) with 5.2% of VAF.

Based on the World Health Organization (WHO) 2016 criteria, he was diagnosed as overt primary myelofibrosis with PNH-clones. The risk stratification of PMF based on Dynamic International Prognostic Scoring System (DIPSS) Plus [19] was DPISS Plus score 5, a high-risk group; age > 65 years, constitutional symptoms such as inactivity and fatigue, hemoglobin < 10 g/dL, circulating blasts 1% and transfusion dependency. Evaluating the symptom burden using the Myeloproliferative Neoplasm Symptom Assessment Form Total Symptom Score (MPN-SAF TSS) [20], the score was 9 showing symptoms of early satiety and problems with concentration. He started treatment with a ruxolitinib dose of 15 mg twice daily with a baseline platelet count of 100 × 10^9^/L or more. Eculizumab treatment for PNH clones did not meet the reimbursement standards of the National Health Insurance Service in our country because there was no evidence for thrombosis, hemolysis, or hemoglobinuria. In the future, eculizumab treatment should be considered depending on the increase in PNH clones and whether hemolysis or thrombosis proceed.

At the follow-up visit two weeks after ruxolitinib treatment, thrombocytopenia progressed with general weakness (Table 1). Therefore, the patient was treated at a reduced dose of ruxolitinib 10 mg twice a day. At the 4th week of ruxolitinib treatment, anemia and thrombocytopenia worsened (Table 1) and MPN-SAF TSS was 20 showing symptoms of fatigue, early satiety, inactivity and problems with concentration. He has temporarily discontinued the treatment with ruxolitinib. One week later, the patient was hospitalized with fever, cough and sputum and was diagnosed with pneumonia. It is now four weeks after withholding of ruxolitinib treatment and the cytopenias have not yet recovered (Table 1).

## 3. Discussion

Our patient showed coexistence of PMF and PNH clones accompanied by *JAK2*, *U2AF1* and *SETBP1* gene mutations. In PMF, high molecular risk mutations include *ASXL1*, *EZH2*, *SRSF2*, *U2AF1Q157* and *IDH1/2*, according to the mutation-enhanced International Prognostic Scoring System (MIPSS-70) [4,5]. The *U2AF1* mutations were observed in 9.3–16% of PMF patients [3,21] and it is known to be associated with poor survival [22,23]. On the other hand, the *SETBP1* mutation is rare in PMF patients (0.3–2.4%) [3,6,24,25], while *SETBP1* was identified in atypical chronic myelogenous leukemia (CML) with a frequency of 32% [24]. One report states that increased *SETBP1* gene expression is associated with a progression from PMF to acute myeloid leukemia (AML) [26]. However, it is still insufficient to conclude the clinical significance of *SETBP1* mutation in PMF patients.

Furthermore, myeloid neoplasm-related gene (*TET2*, *SUZ12*, *U2AF1* and *JAK2*) mutations found in MPN were identified in 83% of PNH patients by NGS [8]. The analysis on hierarchical clonal architecture suggested that such additional mutations arose as a subclone in *PIGA*-mutated cells or prior to *PIGA* mutation [8]. The other study also demonstrated that PNH patients showed multiple somatic mutations along with *PIGA* gene mutation and especially *SETBP1* mutation was also found [10]. Taken together, mutations of *JAK2*, *U2AF1* and *SETBP1* found in our patient were reported associated with either PMF or PNH clones. In PNH, acquiring additional MPN-associated gene mutations might promote clonal expansion of the *PIGA* mutant clone, consequently inducing clinical manifestation of both MPN and PNH [27].

Interestingly, a few cases of coexistence of PMF and PNH have been reported (Table 2) [11,12,14,16]. One recent study reported that PNH clones were found in 2% of MPN patients [15]. The sequence of development of these two clonal diseases or the time points of acquiring these associated mutations has been reported to be variable case by case. According to the previous reports, some patients were initially diagnosed with MPN and PNH together, harboring concomitant MPN associated genes and *PIGA* gene mutations [14,16,17,27]. Another report showed that patients were initially diagnosed with MPN, following eventual evolution of PNH [11,12,13], while other patient was diagnosed as PNH at first and then developed MPN later [12]. Taken together, MPN and PNH association may be attributed to the PNH clone arising either from the MPN-mutated populations or in parallel with the MPN mutated populations and vice versa.

Thrombosis is a common and fatal complication in both MPN and PNH [7,28]. A meta-analysis pooled from 29 cohort studies reported that thrombosis occurred in 20% of patients with MPN [29]. The thrombophilic condition in MPN is caused by quantitative and qualitative changes of blood cells due to the increase in clonal cells of hematopoietic stem cells [28]. Age (>60 or 65 years of age) and a history of thrombosis are independent predictors of thrombosis in MPN patients [28]. *JAK2* V617F was found in 40.9% and 31.5% of patients with Budd Chiari syndrome or portal vein thrombosis, suggesting that MPN is implicated in thrombotic conditions [30]. On the other hand, among MPN patients, thrombotic events were reported at a lower rate in PMF patients than in polycythemia vera or essential thrombocythemia [28].

In PNH, the thrombotic tendency is caused by several factors, while the interplay between complement and coagulation systems plays a significant role [7]. PNH clone size has been proven to be associated with the risk of thrombosis [31]. A PNH case developed from a hemolytic episode to a thromboembolic episode after suffering from PNH disease for 15 years, indicating that the nature of the disease might change with age [32]. Although the main symptom of PNH is caused by intravascular hemolysis, it should note that the risk of thrombosis may increase in advanced age. Our patient was of advanced age at diagnosis, but the follow-up period was short. Thrombotic episodes should be carefully monitored to see if treatment with the anti-complement agent, eculizumab, is required in the future.

In MPN patients with a high risk of thrombosis, useful prognostic and predictable biomarkers are needed. Recently, there have been reports suggesting the potential for diagnosing MPN and predicting thrombosis as a biomarker using liquid biopsy and liquid biopsy-based biomarkers in MPNs [33]. In MPN patients experiencing thrombosis, cell-free DNA, circulating endothelial cells, microparticles (MPs), tissue factors with MPs and procoagulant activity of MPs in peripheral blood were increased and platelet-derived MPs was decreased [33]. When there are complex prognostic factors, such as in this case, biomarkers derived from liquid biopsy have the potential to help predict the prognosis.

Our patient showed coexistence of PMF and PNH clone accompanied by *JAK2*, *U2AF1* and *SETBP1* gene mutations at the first visit to our hospital. However, this patient’s lack of previous clinical history due to a referral from another hospital prevents us from investigating the sequence of these two clonal diseases or the time points of acquiring these mutations. The clinical significance of the coexistence of PMF and PNH clone has not been fully elucidated yet (i.e., how PNH clones could influence MPN phenotype and complication). The complex pathogenesis of these two diseases leaves us with further investigations for clinical significance in a large cohort.

## Figures and Tables

**Table 1:** Laboratory findings overtime.

Laboratory Parameter (Unit)	At Diagnosis	2 Weeks under Ruxolitinib	4 Weeks under Ruxolitinib	4 Weeks Off Ruxolitinib	Reference Range
Hgb (g/dL)	6.0	8.3	6.1	6.6	14.0–16.5
Hct (%)	19.0	26.4	19.3	20.3	42–50
MCV (fL)	84.4	85.2	84.6	87.5	80–96
MCH (pg)	26.7	26.8	26.8	28.6	27–33
MCHC (g/dL)	31.6	31.4	31.6	32.7	33–36
Reticulocyte (%)	2.58	ND	ND	0.81	0.6–2.4
WBC (×10^9^/L)	5.86	5.88	7.44	7.36	4.0–10.0
PLT (×10^9^/L)	128	74	53	53	150–450
LDH (IU/L)	640	537	559	401	106–211
Total bilirubin (mg/dL)	0.88	0.78	0.90	1.22	0.2–1.2
Direct bilirubin (mg/dL)	0.17	0.16	0.18	0.33	0–0.4
AST (IU/L)	20	33	24	16	0–50
ALT (IU/L)	9	27	16	16	0–50
Uric acid (mg/dL)	9.1	9.2	8.8	7.8	2.6–7.6
Haptoglobin (mg/dL)	<8	ND	ND	ND	30–200
EPO (mIU/mL)	189.0	ND	ND	ND	4.3–29.0
Plasma Hgb (g/dL)	4.85	ND	ND	ND	0–5.0
Blood, urine	Negative	ND	ND	ND	Negative
Hemosiderin	Negative	ND	ND	ND	Negative
Direct antiglobulin test (Direct Coombs’ test)	Negative	ND	ND	ND	Negative
Indirect antiglobulin test (Indirect Coombs’ test)	Negative	ND	ND	ND	Negative

Hgb, hemoglobin; Hct, hematocrit; MCV, mean corpuscular volume; MCH, mean corpuscular hemoglobin; MCHC, mean corpuscular hemoglobin concentration; ND, not determined; WBC, white blood cells; PLT, platelets; LDH, lactase dehydrogenase; AST, aspartate aminotransferase; ALT, alanine aminotransferase; EPO, erythropoietin.

**Table 2:** Cases of PMF patients with concurrent paroxysmal nocturnal hemoglobinuria (PNH).

References	Age (yr)/Gender at Dignosis	Presenting Signs and Symptoms	Diagnosis		Genetic Mutation	PNH Clones (% of RBC Granulocytes/Monocytes)	Treatment	Complication	Prognosis
			Init.	F/U (Time)					
Shaheen S.P. 2nd, et al., 2005 [11]	53/M	Splenomegaly, leukocytosis	MPN	PMF PNH (2 yrs)	ND	ND	Hydroxyurea, interferon	Myocardial infarction, precursor B lymphoblastic leukemia	Died for intracranial hemorrhagesepsis
Sugimori C., et al., 2012 [12]	#1: 51/M	Stroke, Budd Chiari syndrome	PMF	PNH (ND)	*JAK2* V617F	13%/99%/ND	Eculizumab	Multiple complications of Budd Chiari syndrome, including esophageal variceal bleeding	Alive
	#2: 65/M	Dark urine, transfusion dependent anemia	PNH	PMF (2 yrs)	*JAK2* V617F	40%/76.7%/ND	Hydroxyurea, anticoagulant, eculizumab	Splenic infarction, portal vein thrombosis	Died for liver failure
	#3: 78/M	Progressive anemia, dark urine	PNH	PMF (1 yr)	*JAK2* V617F	53%/73%/ND	Eculizamab, anticoagulant	Pneumonia	Died for Clostridium difficile infection
Gaidano V., et al., 2017 [14]	72/F	Portal vein thrombosis	Post ET-MF? PNH		*JAK2* V617F	71%/88.6%/86.9%	Hydroxyurea, anticoagulant	None	Alive
Kirito K. 2020 [16]	49/M	Anemia, thrombocythe-mia, dark urine	PMF PNH		*MPL*W515L	ND/99%/99%	ND	ND	ND
Our case	87/M	Progressive anemia, splenomegaly, lymphadenopathy	PNH PMF		*JAK2* V617F *U2AF1Q157* *SETBP1*	3.8%/48.6%/77.2%	Ruxolitinib	Pneumonia	Alive

PMF, primary myelofibrosis; MPN, myeloproliferative neoplasm; yr, year; Init., initial; F/U, follow up; M, male; ND, not determined; F, female; ET, essential thrombocythemia
